# Cytotoxic tau released from lung microvascular endothelial cells upon infection with *Pseudomonas aeruginosa* promotes neuronal tauopathy

**DOI:** 10.1016/j.jbc.2021.101482

**Published:** 2021-12-08

**Authors:** Chung-Sik Choi, Meredith Gwin, Sarah Voth, Claire Kolb, Chun Zhou, Amy R. Nelson, Althea deWeever, Anna Koloteva, Naga S. Annamdevula, James M. Murphy, Brant M. Wagener, Jean-Francois Pittet, Ssang-Taek S. Lim, Ron Balczon, Troy Stevens, Mike T. Lin

**Affiliations:** 1 Department of Physiology and Cell Biology, University of South Alabama, Mobile, Alabama, USA; 2 Center for Lung Biology, University of South Alabama, Mobile, Alabama, USA; 3 Department of Biochemistry and Molecular Biology, University of South Alabama, Mobile, Alabama, USA; 4 Department of Anesthesiology and Perioperative Medicine, The University of Alabama at Birmingham, Birmingham, Alabama, USA

**Keywords:** endothelium, neuron, tau protein, aggregation, infection, BiFC, biomolecular fluorescence complementation, gRNA, guide RNA, HBSS, Hank’s balanced salt solution, PMVECs, pulmonary microvascular endothelial cells, RD, repeat domain, T3SS, type 3 secretion system

## Abstract

Patients who recover from nosocomial pneumonia oftentimes exhibit long-lasting cognitive impairment comparable with what is observed in Alzheimer’s disease patients. We previously hypothesized that the lung endothelium contributes to infection-related neurocognitive dysfunction, because bacteria-exposed endothelial cells release a form(s) of cytotoxic tau that is sufficient to impair long-term potentiation in the hippocampus. However, the full-length lung and endothelial tau isoform(s) have yet to be resolved and it remains unclear whether the infection-induced endothelial cytotoxic tau triggers neuronal tau aggregation. Here, we demonstrate that lung endothelial cells express a big tau isoform and three additional tau isoforms that are similar to neuronal tau, each containing four microtubule-binding repeat domains, and that tau is expressed in lung capillaries *in vivo*. To test whether infection elicits endothelial tau capable of causing transmissible tau aggregation, the cells were infected with *Pseudomonas aeruginosa*. The infection-induced tau released from endothelium into the medium-induced neuronal tau aggregation in reporter cells, including reporter cells that express either the four microtubule-binding repeat domains or the full-length tau. Infection-induced release of pathological tau variant(s) from endothelium, and the ability of the endothelial-derived tau to cause neuronal tau aggregation, was abolished in tau knockout cells. After bacterial lung infection, brain homogenates from WT mice, but not from tau knockout mice, initiated tau aggregation. Thus, we conclude that bacterial pneumonia initiates the release of lung endothelial-derived cytotoxic tau, which is capable of propagating a neuronal tauopathy.

Neurofibrillary tau tangles are hallmark lesions found in the postmortem brains of patients who suffered from Alzheimer’s disease and related dementias. These tangles are intracellular inclusions of hyperphosphorylated and aggregated forms of cytotoxic tau (1) with a routine pattern of spread, often used for classifying the stage of AD severity (2). Emerging evidence indicates these intracellular tau aggregates are transmissible among neurons and cause feed-forward neuronal injury. This tau seed hypothesis suggests that the cytotoxic tau promotes morphological changes to monomeric tau, leading to an expansion of the aggregated protein pool (3, 4). Although the mechanism by which tau seeds convert monomeric tau into pathological species is incompletely understood, this process plays a crucial role in the etiology of tauopathies and the progression of these diseases (5).

Although the mechanism of oligomeric tau propagation is unknown, at least three possibilities have been considered. First, oligomeric tau may be generated within neurons and spread *via* neuronal connectivity in the central nervous system. In this case, tau seeds are released from a presynaptic neuron and taken up in the postsynaptic neuron after receptor binding. Once in the postsynaptic neuron, the aggregated tau seed nucleates monomeric tau, or causes templated fibrillization, forming newly expanded tau aggregates (6, 7, 8). Second, neurons may release aggregated tau into the extracellular space *via* microvesicles, where it disseminates to and is taken up by various cell types, including neurons and glial cells. Once in these cells at sites peripheral to its origin, the cytotoxic tau nucleates monomeric tau and promotes neuroinflammation (9, 10). Third, tau may be generated in peripheral organs and then access the brain. In this case, tau may be transported across the blood-brain barrier or the choroid plexus, where it accesses the cerebrospinal fluid and adjacent neurons (11, 12, 13). Uptake into these neurons could initiate the propagation of tau aggregation. In support of this idea, the introduction of pathological forms of tau and other amyloids into the circulation leads to neuropathology (14, 15), indicating that amyloids in the circulation can access the brain and impair its function. Resolving the mechanisms of tau transmissibility within the natural course of disease remains a major focus of study.

Recently, our group has found that pneumonia elicits the production of cytotoxic tau oligomers within the lung, and more specifically, from lung endothelial cells (16, 17, 18). Lung endothelial cells release cytotoxic tau proteins after *Pseudomonas aeruginosa*, *Klebsiella pneumoniae*, and *Staphylococcus aureus* bacterial infections. The cytotoxic tau variants can be retrieved from the bronchoalveolar lavage fluid, the circulation, and the cerebrospinal fluid of patients and animal subjects with ongoing infection. Their presence in the brain during and after infection corresponds with impaired hippocampal long-term potentiation and learning and memory. Critically ill patients, and especially those with pneumonia, commonly exhibit delirium that progresses to a long-term cognitive deficit, even after their recovery from critical illness (19, 20, 21). We have hypothesized that lung-derived oligomeric tau may be released into the circulation and access the brain, where it contributes to the neurocognitive deficits that are associated with a critical illness (22).

At present, it is unclear whether the lung-derived tau can cause propagation of pathological tau variants, or seeds, in neurons. In the central nervous system, six human pathological tau isoforms have been described, all arising from a single gene (23). These tau isoforms are defined by the presence or absence of two amino-terminal inserts (0N, 1N, and 2N) and three or four microtubule-binding domains (3R and 4R). The smallest isoform is expressed in a 0N3R orientation, whereas the full-length tau is a 2N4R orientation (24). The peripheral nervous system and peripheral organs also express a unique big tau isoform (25, 26); this is a 2N4R isoform that possesses amino-terminal inserts encoded from exons 4A and 6. Notably, all tau isoforms that express the microtubule-binding repeat domain (RD) may have the propensity to form paired-helical filament aggregates in the presence of pathological tau (27). Although we have demonstrated that the lung and lung endothelium express tau, full-length tau has not been cloned. Here, we report four full-length lung endothelial cell tau isoforms, illustrate lung capillary expression of tau *in vivo*, and demonstrate that the infection-elicited endothelial cell tau initiates neuronal tau aggregation.

## Results

**Figure 1:**
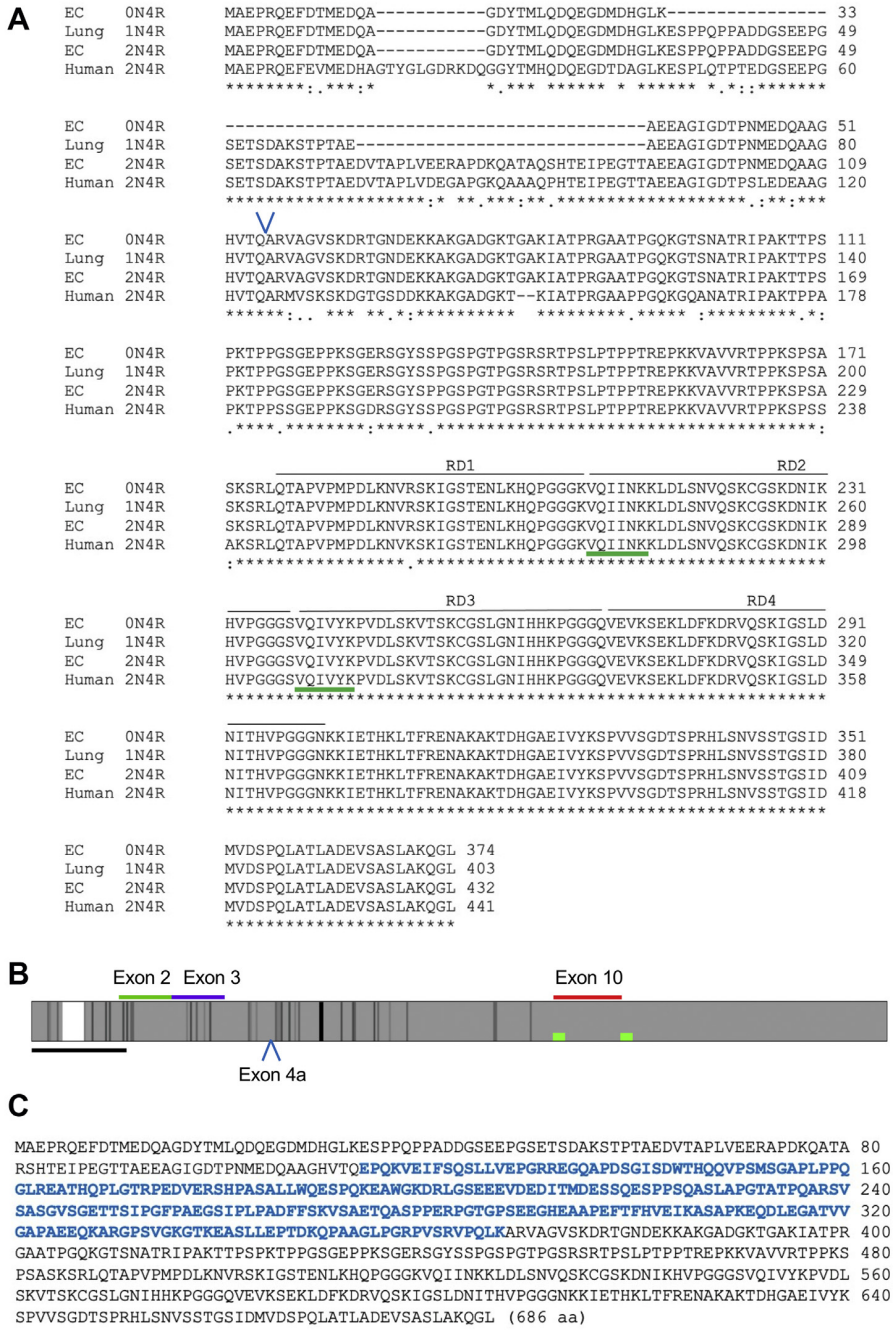
**Endothelial tau isoforms.***A*, four endothelial tau isoforms were cloned directly from rat lung (1N4R) and primary endothelial cell (EC; 0N4R, 2N4R, and the big tau) culture. The amino acid (aa) sequences of the clones are aligned with the human brain’s longest tau isoform, namely the 2N4R. The four repeat domains (RD) that stabilize microtubules are highlighted. The amino acids of rat EC and human 2N4R were compared, and the asterisks indicate identical aa. The *blue caret* indicates the beginning site of additional aa encoded by exon 4a. *B*, the *gray bar* shows aa alignment of cloned and human 2N4R isoforms. The *shades of gray* represent aa matches; *white area* in the N-term represents 11 aa missing in rodents; and *black area* represents two additional aa in the rodent isoform. The *top bars* show the positions of the splice variant exons 2, 3, and 10 and the aggregation domains VQIINK and VQIVYK (*red*). The location of exon 4a that is expressed by big tau is indicated by the *caret*. The *bottom scale bar* represents 50 aa. Note that the c-termini of endothelial tau isoforms including the RD regions are 99% identical to that of the human tau aa. *C*, big tau sequence with the aa encoded by exon 4a shown in *blue*. Endothelial big tau is composed of 686 aa.

To generate and identify the cDNA clones encoding tau isoforms in rat lung, we performed RT-PCR using rat lung tissue and primary pulmonary microvascular endothelial cells (PMVECs). Our results identified three rat lung tau isoforms that are reminiscent of the human tau isoform, and a big tau isoform known to be expressed in the periphery (Fig. 1). Aligning our cDNA clones with the longest human tau isoform, that is, the 2N4R [both N-termini and four repeat domains], our results indicated that rat lung expresses 0N4R-, 1N4R-, and 2N4R-isoforms, and a big tau isoform. Notably, the rodent and human *MAPT* genes differed mainly in the N-termini, whereas the C-termini containing the four microtubule-binding RDs were nearly identical. The big tau isoform included an additional 254 aa, encoded by exon 4a (Fig. 1*C*).

**Figure 2:**
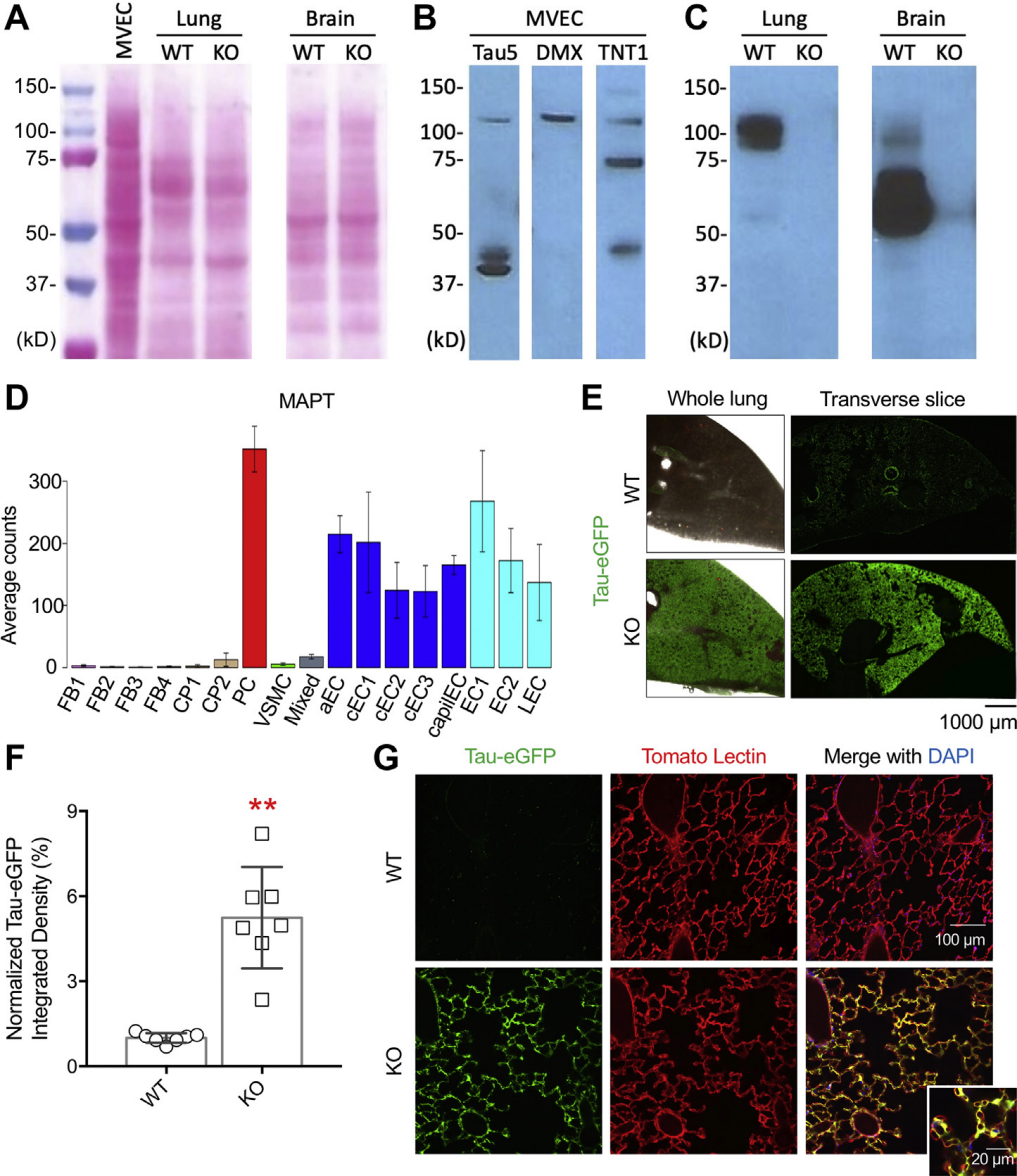
**Lung capillaries express tau.** Lung microvascular endothelial cell (MVEC) and lung and brain lysates from WT or tau KO mice were prepared for Western blotting. *A*, ponceau S staining shows the amount of protein loaded. *B*, endothelial tau was probed with different tau antibodies, including the Tau5, 1DM9X (DMX), and TNT1 antibodies. *C*, WT mouse lung and brain express tau, which is missing in the tau KO mice. The membrane was probed with the tau C-terminal antibody D1M9X. All the samples were run on the same gel; the big gap between lung and brain lanes indicates image splice (*A* and *C*). *D*, *MAPT* gene expression in adult mouse lung vascular and perivascular cells. The figure, recreated using an online scRNAseq database, shows averaged *MAPT* expression in various lung cell types. *E*, representative images showing fluorescence in lungs obtained from WT (*top panels*) and tau KO (*bottom panels*) mice. The whole lung images were captured with a 4× objective, and the transverse slice images were captured using a 20× objective and the images were stitched together. *F*, quantification of tau-eGFP expression in KO mouse. Integrated green fluorescence density was normalized to that of the WT mouse. KO mice expressed significantly higher fluorescence than WT (*p* < 0.001; one-tail *t* test). Each data point represents an animal. ∗∗*p* < 0.001. *G*, representative images showing fluorescence in gelatin-agarose-infused lung slices. 800 μm thick lung sections were cut, and the alveolus and capillaries were imaged by a 25× objective on a multi-photon microscope. The B6.129S4(Cg)-*Mapt*^*tm1(EGFP)Klt*^/J mouse harbors a tau knockout with an eGFP insertion in exon 1 of the tau locus so that tau-expressing cells can be visualized. The endothelium was labeled with tomato lectin (Lycopersicon esculentum; *red*) injected through the circulation, and airways of WT and KO mice were then filled with gelatin and agarose, respectively. Cell nuclei were stained with DAPI. Lung capillaries exhibited *green* (eGFP) and *red* (tomato lectin) fluorescence, indicating that they express endothelial tau under basal conditions. The *bottom inset* image shows an enlarged area to highlight the endothelial cells that encapsulate capillaries (*small circles*) surrounding alveolae (*large circle* and *empty space*) express tau reporter and are labeled with tomato lectin. a, arterial; c, continuum; capil, capillary; CP, cartilage perichondrium; EC, endothelial cells; FB, vascular fibroblast-like cells; L, lymphatic; 1,2,3,4, subtypes; PC, pericytes; VSMC, vascular smooth muscle cells.

We next examined the tau expression in rat PMVECs and in WT and tau knockout mouse lung and brain using Western blotting (Fig. 2). We probed the MVEC membranes with several tau-selective antibodies, including the Tau5 (detects tau; epitope upstream of RD1), D1M9X (detects tau; C-terminus epitope), and TNT1 (detects pathological tau; N-terminus epitope) antibodies. Our result showed overlapping and distinct bands at 110, 75, and ∼40 kDa (Fig. 2*B*). Interestingly, the predominant tau isoform found in the lung had a molecular weight of ∼100 kDa, whereas the predominant brain tau isoform was at ∼60 kDa (Fig. 2*C*).

Our Western blot results are consistent with the studies that have previously indicated that lung endothelium expresses tau (28, 29). These findings are also supported by RNAseq results. As shown in Figure 2*D*, created using the online scRNAseq database and tool (http://betsholtzlab.org/VascularSingleCells/database.html), the *MAPT* gene is expressed in various cell types in adult mouse lung. In the current study, we confirmed the expression of tau in lung capillaries *in vivo*. The B6.129S4(Cg)-*Mapt*^*tm1(EGFP)/Klt*^/J mouse harbors a tau knockout with an eGFP insertion in exon 1. Thus, eGFP fluorescence serves as a reporter for tau-expressing cells. As seen in Figure 2*E*, the lung parenchyma *ex vivo* expresses tau-eGFP, consistent with abundant tau-expressing cells. The eGFP-tau signal colocalized with tomato lectin, an indicator of the endothelium, and was particularly abundant in capillaries (Fig. 2, *F* and *G*) (30). The images obtained from brain slices also revealed extensive tau-eGFP expression in cells, consistent with the expected neuronal tau expression (data not shown).

We have previously shown that Gram-positive and Gram-negative organisms that cause pneumonia trigger the release of cytotoxic tau variants from lung endothelium, as quantified using Western blotting with antibodies selective for various tau species (*e.g.*, TOC1, TauC3, Tau5, and T22) (17, 31). In a recent study, we determined the ‘bioactivity’ of endothelium-derived tau and amyloid species released from lung endothelium after infection with *P. aeruginosa* strain variants (32). We studied virulent (PA103 and ExoY) and nonvirulent (ΔPcrV and K81M) *P. aeruginosa* strains. Specifically, ΔPcrV lacks a functional type 3 secretion system and is a control for the PA103 strain; K81M is an ‘ExoY-enzyme dead’ control for the ExoY strain. Our results show that when exposed to *P. aeruginosa* bacteria, lung endothelial cells produce and release the Tau5 and T22 immunoreactive tau species into cell media. Notably, the bioactivity of these tau species is dependent upon the bacterial virulence (32, 33, 34, 35).

**Figure 3:**
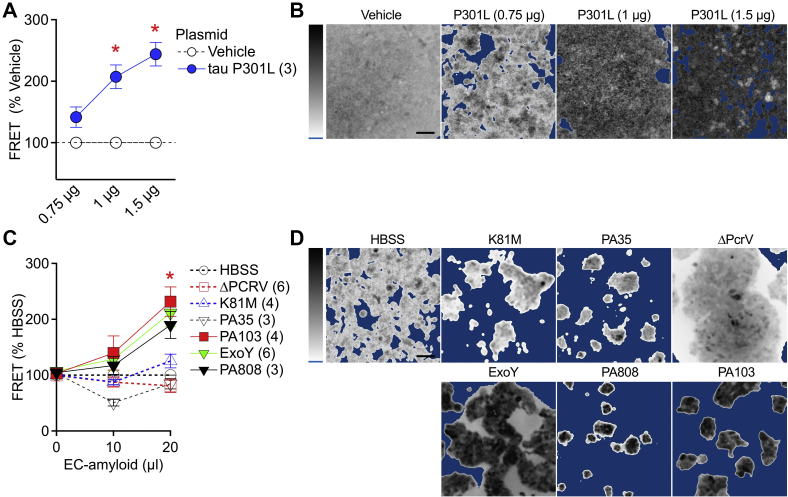
**Endothelium-derived tau nucleates neuronal tau.** HEK293 FRET reporter cells that stably express the RD-CFP and RD-YFP were used. *A*, summary of normalized FRET efficiency in cells transfected with the indicated amount of tau P301L plasmids. Each group is plotted as mean ± S.E.M. ∗*p* = 0.030 and 0.0048 *versus* vehicle control for 1 μg and 1.5 μg, respectively (ANOVA, followed by Dunnett’s test). *B*, representative FRET signal micrographs after plasmid transfection. The *dark areas* indicate a high FRET signal, whereas *lighter areas* indicate a low signal. *Blue* is the background and indicates no cell or FRET signal. Vehicle was OPTI-MEM without DNA plasmid. *C*, summary of normalized FRET efficiency in cells transfected with endothelium-derived tau. Endothelium-derived tau was prepared from endothelial media after the cells were exposed to the indicated *Pseudomonas aeruginosa* strains. Each group is plotted as mean ± S.E.M. *Asterisk* denotes statistical significance *versus* vehicle control (ANOVA, followed by Dunnett’s test; PA103 *p* = 0.0013; ExoY *p* < 0.001; and PA808 *p* = 0.043). *D*, representative FRET signal micrographs after transfection. Vehicle HBSS was collected from endothelial medium, but the cells were not infected with *P. aeruginosa*. The numbers of experimentation (n) are indicated in *brackets*. Vertical colorimetry shows FRET signal strength; *blue* = background. The scale bars represent 100 μm. HBSS, Hank’s balanced salt solution.

Because infection elicits endothelial production and release of tau into the supernatant, we determined whether the supernatant induces neuronal tau aggregation. We used the previously established HEK293 cells that stably express the tau-RD and CFP or YFP in a FRET-based assay (15, 36). We first determined the FRET signal in these cells 24 h after they were transiently transfected with a DNA plasmid containing the full-length tau that harbors the P301L mutation known to cause pathological tau aggregation (37). The FRET signal was normalized to the control vehicle of each study (see FRET measurement in Experimental procedures). Our results confirmed that increasing the P301L expression increased FRET (Fig. 3, *A* and *B*). Next, endothelial cells were exposed to various *P. aeruginosa* strains, and the supernatant was collected, centrifuged, and filter sterilized (see supernatant preparation in Experimental procedures). HEK293 cells were treated with the supernatants, and the FRET signals were determined after 24 h (Fig. 3, *C* and *D*). The supernatant from endothelial cells exposed to virulent *P. aeruginosa* strains/mutants (*i.e.*, ExoY, PA808, and PA103) increased the FRET signal in HEK293 cells. In comparison, the supernatant collected from endothelial cells exposed to nonvirulent *P. aeruginosa* strains/mutants (*i.e.*, K81M, PA35, and ΔPcrV) did not increase the FRET signal in HEK293 cells. These results indicate that *P. aeruginosa* virulence factors, such as the type III secretion system effectors, promote endothelial cell release of factors, such as the endothelial tau, that initiate tau aggregation in naïve (*i.e.*, uninfected) cells.

**Figure 4:**
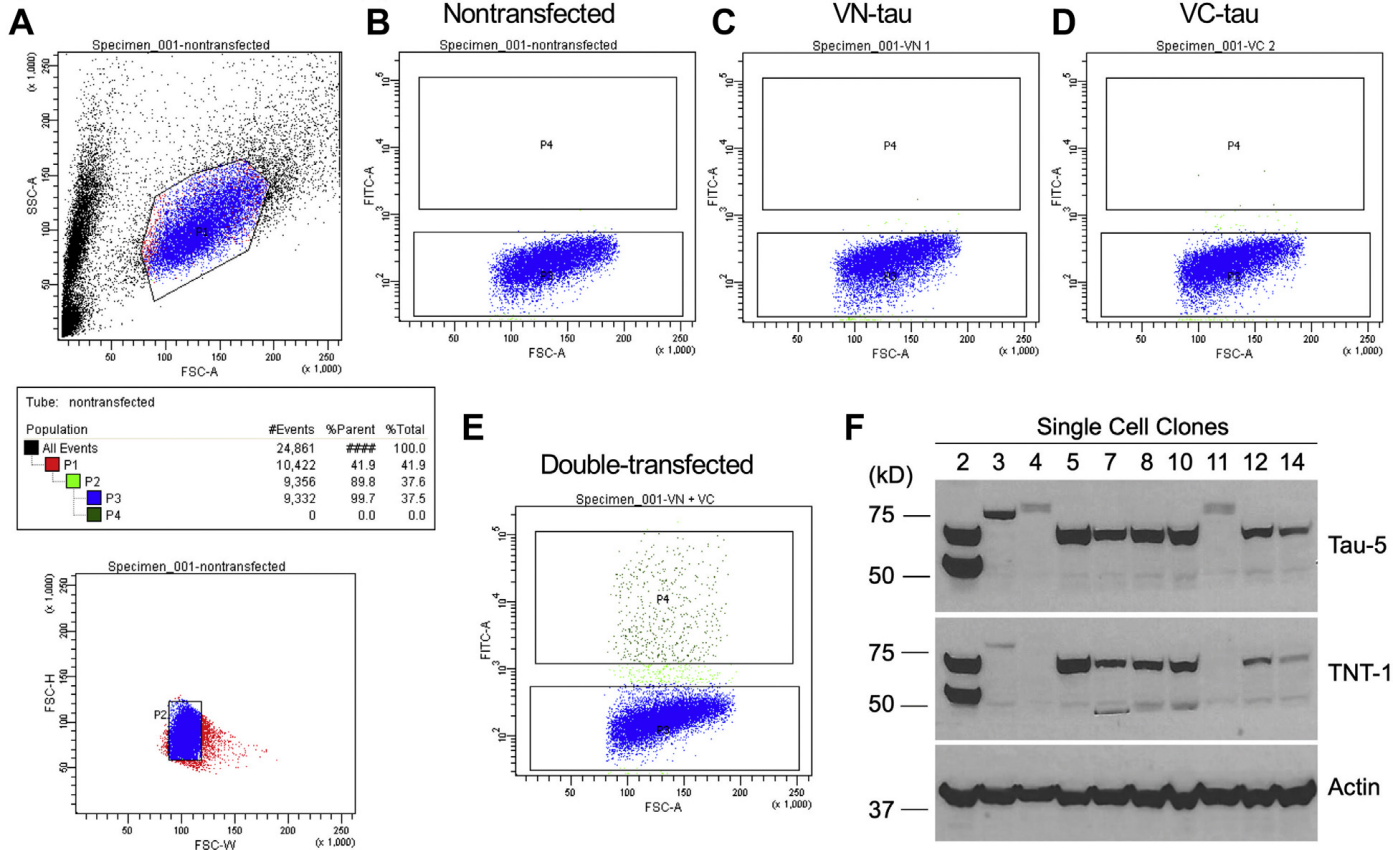
**Generation of stable BiFC reporter single-cell clones using fluorescence-activated cell sorting.** HEK293 cells were transfected with VN-tau, tau-VC, or both VN-tau and tau-VC plasmids, followed by antibiotic selection. *A*, gating strategy using flow cytometry to isolate single HEK293 cells. *B*, control cells not transfected with plasmid show no fluorescence (*bottom box*). *C*–*E*, gating and fluorescence signal in cells transfected with VN-tau (*C*), tau-VC (*D*), or both VN-tau and tau-VC (*E*). Double-transfected tau cells show increased fluorescence signal (*top box*). *F*, representative Western blot quantification of the amount of VN-tau and tau-VC in single-cell clones. Cell clones (*e.g.*, clone 2) expressing a similar amount of VN-tau (*upper band*) and tau-VC (*lower band*) were further expanded and used for BiFC studies. BiFC, biomolecular fluorescence complementation.

We verified these results using a biomolecular fluorescence complementation (BiFC) assay (38). In this assay, we used the longest neuronal 2N4R tau isoform. To test this idea, HEK293 cells were transiently transfected with two plasmids encoding for the following: (1) the N-terminal 177 amino acids of the Venus fluorescent protein (Vn, 173 aa) followed by tau; and (2) tau followed by the C-terminus of the Venus protein (Vc, 155 aa). Two days after the transfection, we exposed the cells to the endothelial supernatant containing infection-elicited tau and determined the BiFC signal 24 h later. Although our initial results looked promising, we noticed that the BiFC intensity varied from study to study, likely because of the transient transfection efficiency (data not shown). Thus, we created a HEK293 BiFC reporter cell line that stably expressed the BiFC probes (Fig. 4). HEK293 cells were transfected with Vn-tau, tau-VC, or both plasmids. Antibiotic-resistant cells were expanded and single-cell sorted with flow cytometry (see Experimental procedures). Figure 4 shows the cell-gating strategy. Notably, double-transfected cells exhibit higher fluorescence, compared with the cells transfected with Vn or Vc alone (Fig. 4, *C*–*E*). Single-cell clones were further expanded, and the cells that expressed equal amounts of Vn-tau and tau-Vc proteins were verified by Western blotting (*e.g.*, cell clone #2 in Fig. 4*F*); these cells were used for the subsequent BiFC studies.

**Figure 5:**
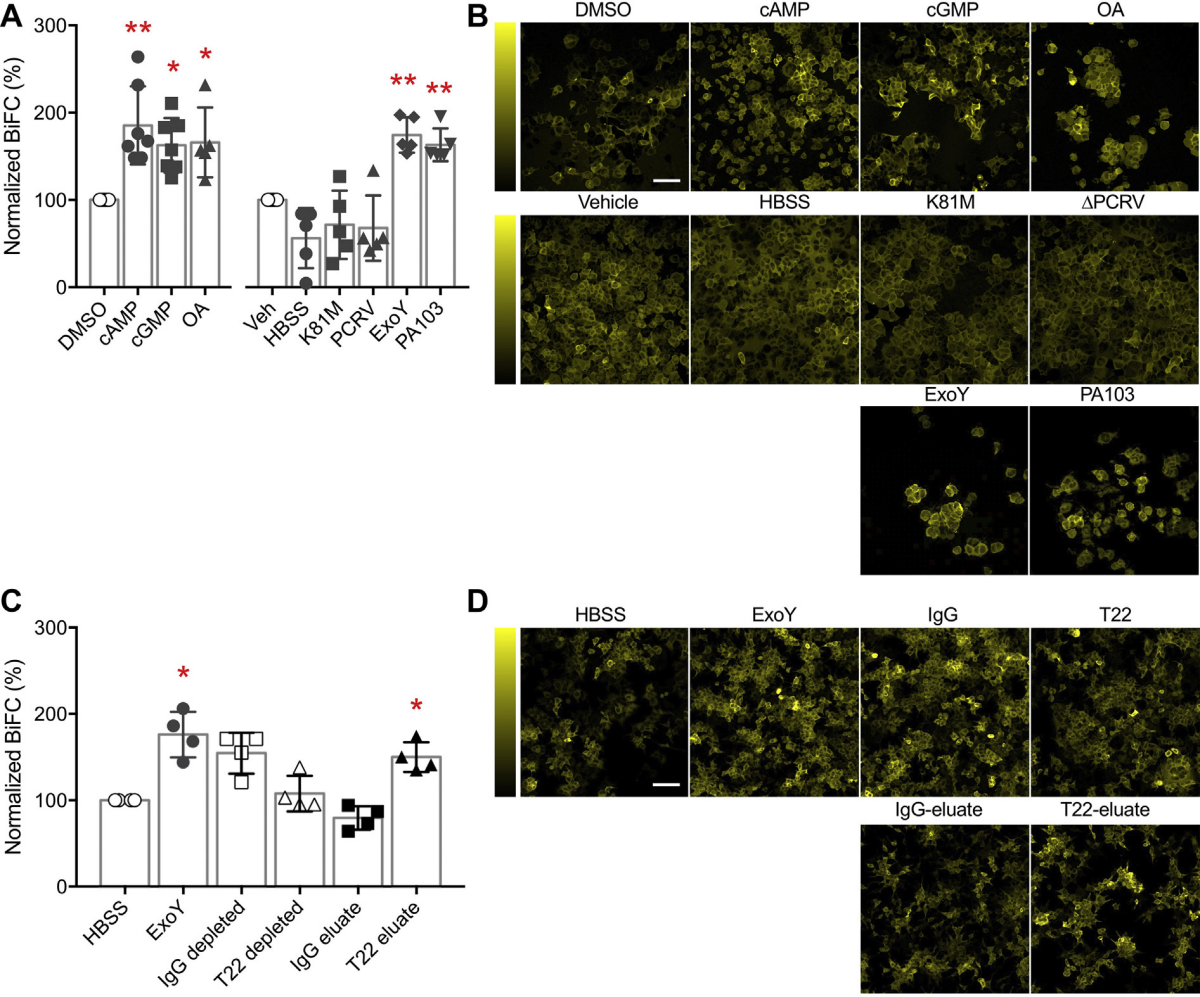
**Endothelium-derived tau nucleates full-length neuronal tau.***A*, summary of normalized BiFC signal after the indicated treatments. *Left*, positive control studies included treatment using a membrane permeable form of cAMP or cGMP and okadaic acid (OA). BiFC signal was normalized to DMSO vehicle control. Statistical comparison was performed using ANOVA and Dunnett’s *post hoc* tests; the *asterisks* denote statistical significance *versus* DMSO (cAMP *p* = 0.0012, n = 7; cGMP *p* = 0.016, n = 7; and OA *p* = 0.018, n = 5). (*right*) Endothelium-derived tau transfection studies were normalized to the vehicle (Veh, water). HBSS cell medium from endothelial cells was not exposed to *Pseudomonas aeruginosa*. Statistical comparison was performed using ANOVA and Dunnett’s *post hoc* tests; the *asterisks* denote significance *versus* Veh (ExoY *p* = 0.0016; PA103 *p* = 0.0078; n = 5). *B*, representative BiFC fluorescence micrographs. *Yellow* fluorescence emitted from the Venus protein indicates neuronal tau aggregation. Vertical colorimetry shows BiFC signal strength. The scale bar represents 100 μm. *C*, summary of BiFC signal after the indicated treatments, normalized to HBSS control. ExoY-infected endothelial supernatant (ExoY) was used as a positive control. ExoY immunodepleted with an IgG antibody (IgG depleted) or the T22 antibody (T22 depleted) and the eluates from the respective antibodies were applied to the BiFC cells. Statistical comparison was performed using ANOVA and Dunnett’s *post hoc* tests; the *asterisks* denote significance *versus* HBSS (ExoY *p* = 0.030; T22 eluate *p* = 0.029, n = 4). IgG depleted control showed a nonsignificant trend of elevated BiFC signal (*p* = 0.054, n = 4). *D*, representative BiFC fluorescence micrographs. The strength of *yellow* fluorescence indicates neuronal tau aggregation. Vertical colorimetry shows BiFC signal strength. The scale bar represents 100 μm. BiFC, biomolecular fluorescence complementation; HBSS, Hank’s balanced salt solution.

In a positive-control study, we first verified the BiFC signal by treating cells with the membrane-permeable (8-pCPT) form of cAMP and cGMP, okadaic acid, and a DMSO vehicle control. Cyclic nucleotides activate protein kinases, and okadaic acid inhibits protein phosphatases, all of which lead to tau phosphorylation, aggregation, and enhanced BiFC. Consistent with a previous report (39), our results showed that these treatments increased the BiFC signal, normalized to the DMSO control (Fig. 5). Next, we treated the cells with endothelial supernatant that was collected after *P. aeruginosa* infection, and we quantified the BiFC signal 24 h later. In this set of studies, BiFC signals were normalized to vehicle (transfection medium) control. The supernatant from ExoY- and PA103-infected cells significantly increased the BiFC signal, whereas the supernatant from HBSS- (Hank’s balanced salt solution; vehicle control), K81M-, and ΔPcrV-treated cells did not (Fig. 5, *A* and *B*).

Although these tau aggregation findings are consistent with the results obtained using the FRET assay, our previous studies have further illustrated that neutralizing tau oligomers using the T22 antibody removes cytotoxins generated by endothelial cells (33, 34). Thus, we tested whether immunodepletion using the T22 antibody neutralizes the endothelial supernatant’s capability to nucleate neuronal tau. For this set of studies, ExoY-infected endothelial supernatant was used as a positive control, and it increased the BiFC signal by 176% (Fig. 5, *C* and *D*). ExoY-infected supernatant was immunodepleted using either the T22 or a control IgG antibody. Immunoreactive tau oligomers were then eluted from the T22 or IgG antibody-bead complex using a high-salt solution. The salt was removed by dialysis against HBSS, and the antibody bound to tau oligomers was denatured by boiling. The resulting fractions and the immunodepleted fractions were applied to the BiFC cells for neuronal tau aggregation quantification. Our results showed that T22 immunodepletion removes the endothelial tau ‘seeds’, whereas the T22 eluate contains the endothelial tau species that can induce neuronal tau aggregation. Thus, these results suggest that virulent strains/mutants of *P. aeruginosa* elicit the release of endothelial tau oligomers into the supernatant, and these pathological variants promote neuronal tau aggregation.

**Figure 6:**
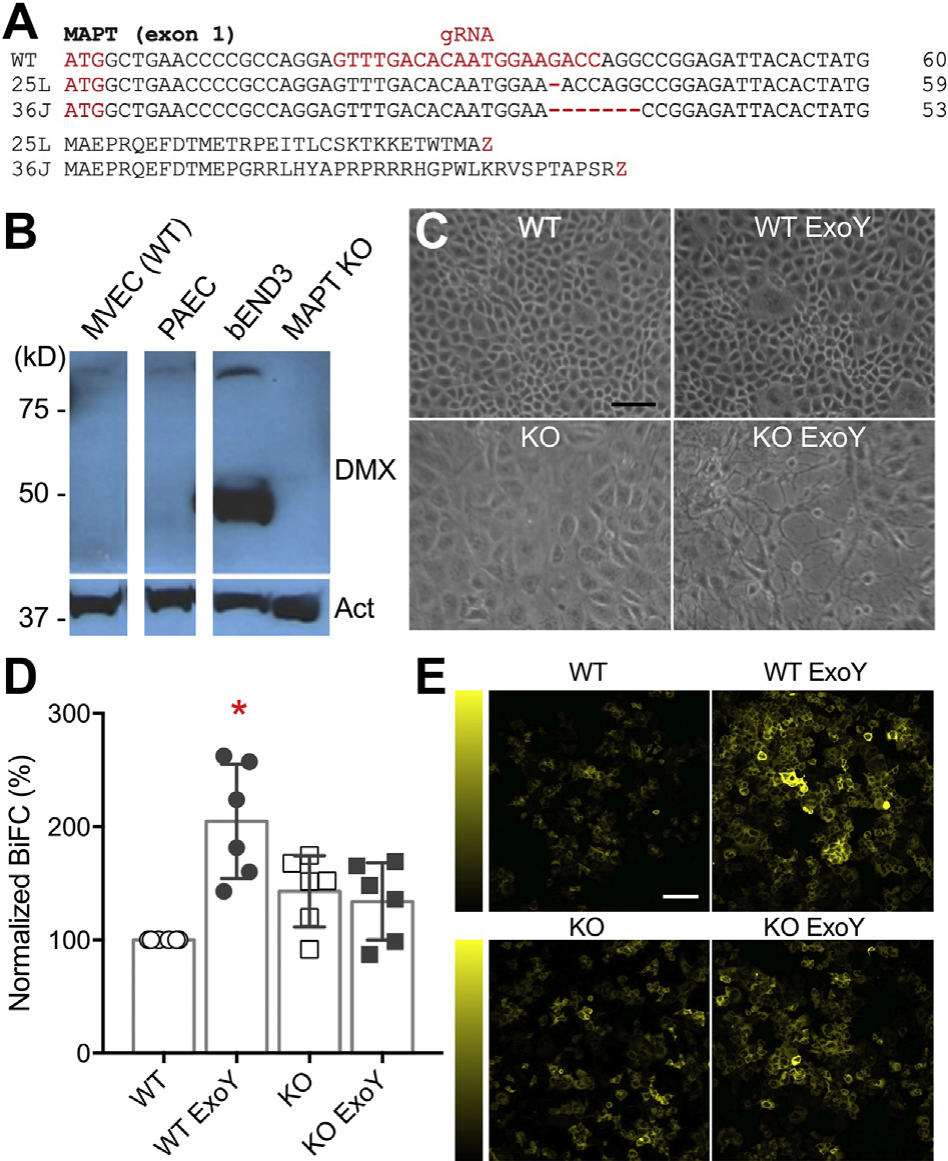
***MAPT* knockout rat endothelial cells do not nucleate neuronal tau.***A*, WT and *MAPT* KO DNA and the expected amino acid sequence alignment with guide RNA (gRNA) targeting exon 1. The guide RNA targeting sequence, frameshift mutation and created termination, and the ATG start codons are in *red*. *B*, Western blot analysis of tau from WT endothelial cells and a *MAPT* KO cell clone. MV (pulmonary microvascular), PA (pulmonary artery), and bEND3 (mouse brain microvascular) endothelium. The blot was probed with the tau C-terminal antibody D1M9X (DMX). *MAPT* KO clones were generated from rat MVEC and do not express tau. Act is actin, which serves as a loading control. All the samples were run on the same gel; the gaps indicate image splices. *C*, cell morphology and gap formation in the absence (*top*) or presence of *Pseudomonas aeruginosa* (3 h, *bottom*). Please note that *MAPT* KO clone showed an enlarged cell surface, and that *MAPT* KO cells showed increased gap formation in *P. aeruginosa*. *D*, summary of normalized BiFC signal after the reporter cells were transfected with supernatant collected from WT or *MAPT KO* endothelium, with or without *P. aeruginosa* (ExoY strain) infection. The *asterisk* denotes statistical significance *versus* WT control (ANOVA, followed by Dunnett’s test; ExoY *p* = 0.011; n = 6). *E*, representative BiFC fluorescence micrographs. *Yellow* fluorescence emitted from the Venus protein indicates neuronal tau aggregation. Vertical colorimetry shows BiFC signal strength. The scale bars represent 100 μm. BiFC, biomolecular fluorescence complementation; MVECs, microvascular endothelial cells.

To examine whether an infection-elicited endothelial tau variant is necessary to induce neuronal tau aggregation, we generated tau KO endothelial cell lines using CRISPR-Cas9 gene-editing technique (Fig. 6). *MAPT* deletion was verified by RT-PCR, immunoblot, and sequencing. The results indicated that two single-cell KO clones were generated by frameshift, resulting in early termination (Fig. 6, *A* and *B*). Notably in the absence of tau, cell morphology (*e.g.*, cell size) and the response to ExoY infection differed from that of the WT. KO cells appeared larger, which was surprising considering that stable microtubules provide the cytoskeleton that is necessary for the cells to spread. After the exposure to ExoY, gap formation among WT cells started to emerge at about 4 to 5 h, which is consistent with our previous studies (17, 32). However, gaps started to appear within 1 to 2 h post ExoY infection in the KO cells (Fig. 6*C*). Next, we collected endothelial supernatant from WT and KO cells, in the absence or presence of ExoY. We treated BiFC reporter cells with the collected supernatant and quantified the signal 24 h later. In this set of studies, BiFC signals were normalized to the uninfected WT cells. Consistent with what was previously shown, ExoY-infected WT cells significantly increased the BiFC signal. In comparison, the supernatant from infected or uninfected *MAPT* KO cells did not increase the BiFC signal (Fig. 6, *D* and *E*).

**Figure 7:**
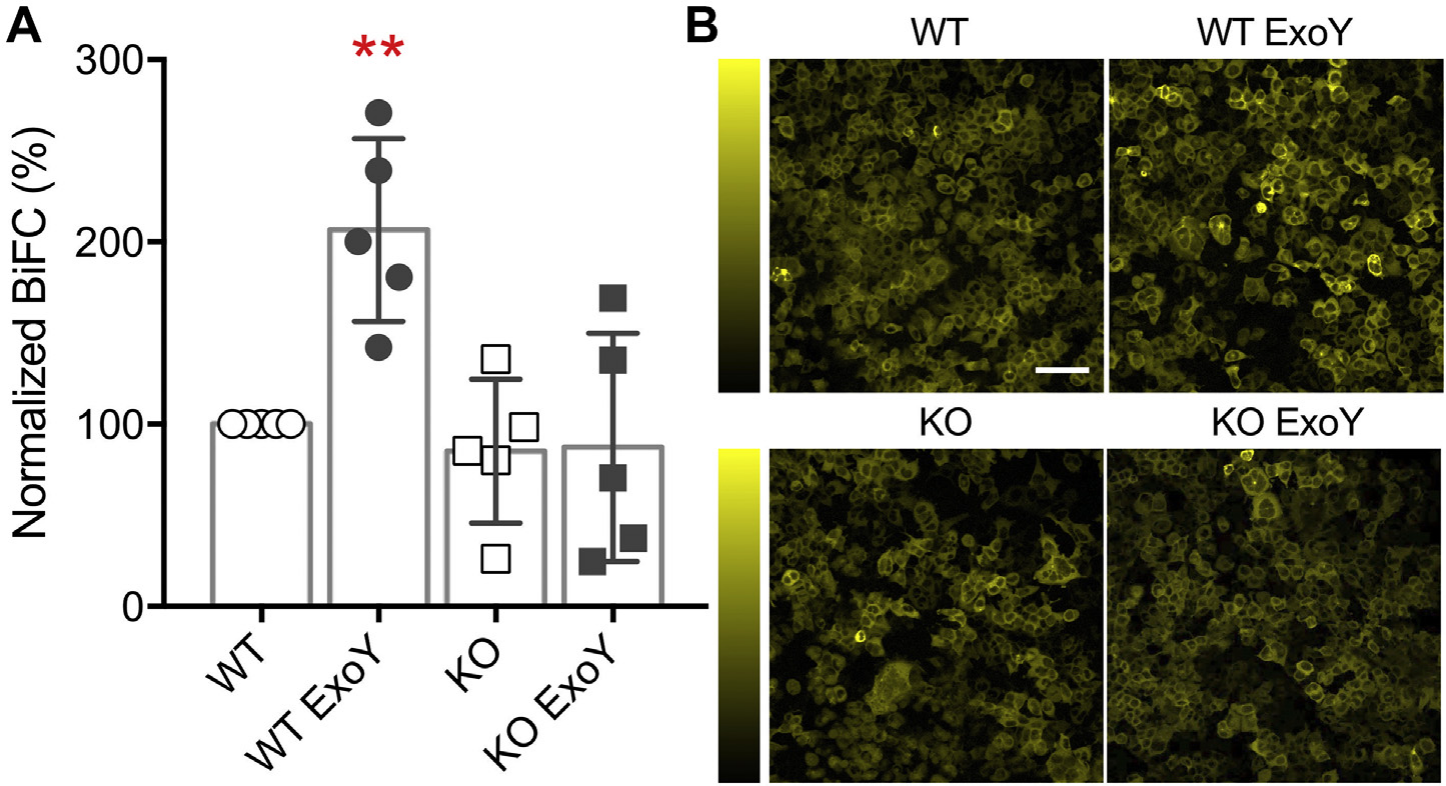
**Lung infection induces neuronal tau seeds in the brain.***A*, summary of normalized BiFC signal after the HEK293 reporter cells were transfected with homogenates prepared from the brain of WT or tau KO mice. The mice were not infected or infected with the *Pseudomonas aeruginosa* ExoY strain. Statistical comparison was performed using ANOVA and Dunnett’s *post hoc* tests; the *asterisks* denote significance (*p* = 0.0046 *versus* WT; n = 5 mice). *B*, representative BiFC fluorescence signal micrographs obtained after the indicated treatments. *Yellow* signal is the fluorescence emitted from the Venus protein and indicates tau aggregation. Vertical colorimetry shows BiFC signal strength. The scale bar represents 100 μm. BiFC, biomolecular fluorescence complementation.

To study whether lung infection increases pathological tau variants in the brain, we instilled ExoY (1 × 10^5^ CFU) into the airways of WT and tau KO mice. Forty-eight hours after the primary infection, we obtained mouse brains and determined whether they are capable of inducing neuronal tau aggregation using the BiFC assay. The brains were homogenized, and 5 μg of protein was suspended in a salt solution and exposed to the HEK293 cells. BiFC signals were determined 24 h after transfection, and the values were normalized to the uninfected WT brain homogenate (Fig. 7). Our results showed that after ExoY infection, WT brains contain pathological tau that can induce neuronal tau aggregation. Notably, this pathological tau species was absent in tau KO brains, with or without infection. Thus, lung infection leads to the presence of cytotoxic tau in the cerebral circulation or within the brain, and it can cause the propagation of neuronal tau aggregation.

## Discussion

We have previously reported that pneumonia causes the generation of cytotoxic tau variants, which accumulate in the bronchoalveolar lavage fluid, the blood, and the brain of patients and animal subjects with infection (18, 33, 34, 40). These cytotoxic tau variants can be produced within the lung, however, whether the lung-derived cytotoxic tau can cause neuronal tau aggregation as a mechanism of disease propagation has not been determined. In this study, we have described a transcellular model in which we demonstrate the tau of endothelial origin initiates neuronal tau aggregation. We first showed that pulmonary endothelial cells express at least four tau isoforms, each of which possesses the aggregation domains. Endothelial infection with virulent *P. aeruginosa* strains/mutants promotes the release of cytotoxic tau variants, and we demonstrated that the supernatant from cells infected with these bacteria seeds neuronal tau aggregation. The endothelial-derived tau causes the RD-tau aggregation, as determined using a well-established cellular FRET-based assay system. Using a reporter cell clone that stably expresses similar amounts of Vn-tau and tau-Vc fluorescent probes, we demonstrated that endothelial tau causes aggregation of the longest (*i.e.*, 2N4R) neuronal tau isoform in a BiFC assay. We then extended this method to document that lung infection causes the accumulation of tau variants within the brain that can cross-seed neuronal tau aggregation. These findings support the infectious tauopathy hypothesis of neurocognitive dysfunction in the post-ICU syndrome (16, 17, 18, 32, 33, 34, 35).

Both Gram-positive and Gram-negative bacteria elicit endothelial production and release of tau from the lung. Notably, in the case of *P. aeruginosa*, bacterial virulence factors are required for the production of an endothelial-derived tau that impairs long-term potentiation and animal learning and memory (33, 34, 35). More specifically, *P. aeruginosa* virulence is attributable to the type 3 secretion system (T3SS) and its associated exoenzymes, which are injected into host cells (32). Here, we tested three T3SS-competent *P. aeruginosa* strains/mutants, including PA103, ExoY, and PA808. PA103 uses exoenzymes T and U, whereas ExoY is engineered to solely introduce exoenzyme Y into host cells. PA808 is a clinical strain isolated from an ICU pneumonia patient; this strain possesses exoenzymes S, T, and Y. All three of these strains activated the endothelium to release a tau variant capable of promoting tau aggregation. Controls for this study included PcrV, K81M, and PA35 strains/mutants. ΔPcrV lacks a functional T3SS, and although it possesses exoenzymes T and U, the exoenzymes cannot be introduced into host cells. K81M is a bacterial mutant that has a functional T3SS and introduces a catalytically dead ExoY into the host cell. PA35 is a clinical isolate from an ICU patient that does not possess the T3SS. None of these nonvirulent strains caused an endothelial release of tau variant capable of inducing tau aggregation. Thus, the presence of a functional T3SS and enzymatically active effectors are important determinants of tau’s bioactivity.

We have identified three tau isoforms in rodent pulmonary endothelium that are similar to the 0N4R, 1N4R, and 2N4R isoforms in neurons (∼37–45 kDa). Previous studies have shown tau expression in peripheral tissues and other cell types (26, 41). Interestingly, peripheral organs, including the lung and peripheral nerves, express a ‘big’ tau isoform (∼100 kDa) that is not found in the brain (25, 42, 43). We also found big tau expression in lung endothelial cells. In our previous studies, we have reported that lung endothelium produces and releases several tau species that are detectable at ∼37, ∼55, ∼75, and ∼100 kDa. In the current study, our Western blot result showed ∼40, ∼75, and ∼110 tau bands in PMVECs using the specific tau antibodies Tau5, D1M9X, and TNT1 (Fig. 2*B*). Consistent with this result, our Western blotting results further showed an ∼100 kDa tau band across endothelial cells from various vascular origins using the C-terminal D1M9X antibody, and this high molecular weight tau band was absent in the *MAPT* KO cells (Fig. 6*B*). In our previous studies, we have attributed the higher molecular weight taus to the isomerization of ‘small’ tau isoforms; however, results from the current study suggest the high molecular weight band may also be a big tau isoform. Thus, lung endothelium expresses at least four tau isoforms.

It is currently unclear whether lung expresses the 3R tau isoforms, and we have yet to fully resolve posttranslational modifications of the tau species released by the endothelium after bacterial exposure. It is notable that knocking out the rat endothelial *MAPT* gene results in bigger cells that are also more prone to infection-induced injury (*i.e.*, gap formation in Fig. 6*C*). Because endothelial tau is released into the cell medium after *P. aeruginosa* infection, these morphological results suggest that endothelial tau plays an important role in barrier integrity maintenance in response to bacterial infection (44).

The tau protein has conserved regions that are crucial for aggregation, including two c-terminal hexapeptide sequences found within the R2 (275-VQIINK-280) and R3 (306-VQIVYK-311) repeat domains. These sequences represent minimal sequences necessary and sufficient for β-pleated sheet formation, and they have been engineered into a FRET-based cellular reporter (45, 46). As seen in Figure 1, rodent and human tau proteins have an identical amino acid composition in the c-terminal region that contains the RD and aggregation domains. The FRET-based and BiFC assays are comprised of the RD aggregation region and the full length (2N4R) tau, respectively. Our results illustrate that only the virulent bacteria-elicited endothelial tau caused neuronal tau aggregation both in the FRET-based and BiFC assays. We have previously shown that the cytotoxicity in endothelial supernatant is immunoneutralized with the T22 antibody. Our results showed that T22 immunodepletion removes endothelial tau seeds, rendering the supernatant incapable of nucleating neuronal tau aggregation. Further, our previous studies have shown that nonvirulent *P. aeruginosa* strains also elicit endothelial tau release, as detected by Western blotting in our previous studies (17, 18); however, these tau species possess antimicrobial bioactivity and are not cytotoxic to host cells (32), and here, they did not nucleate neuronal tau. The host-pathogen mechanisms responsible for the production of pathological *versus* nonpathological tau variants are still poorly understood. Further studies are warranted to fully elucidate the underlying mechanisms.

We examined whether lung infection leads to a cytotoxic tau burden in the brain, and further, whether this tau is sufficient to promote neuronal tau aggregation. We instilled the *P. aeruginosa* ExoY strain into the airways of WT and tau KO mice and determined the presence of pathological tau ‘seeds’ 48 h after infection. The brains from uninfected WT animals were unable to provoke neuronal tau aggregation, whereas the brain from infected WT mice contained pathological tau seeds. These findings are consistent with our recent studies, demonstrating that pneumonia initiates a tauopathy in the brain. Remarkably, the brains from both uninfected and infected tau KO animals were unable to provoke neuronal tau aggregation. We have previously shown that tau oligomers can be detected in the sarkosyl-precipitated brain homogenate 7 days after *P. aeruginosa* lung infection (34). We can also detect pathological tau oligomers in rodents’ plasma, heart, and brain as soon as 48 h after lung infection (22). These results suggest that after lung infection, endothelium produces and releases pathologic tau variants into blood, and through blood circulation, these variants disseminate to the brain. It is noteworthy that the brain homogenates prepared in this study for BiFC quantification may include pathological tau oligomers in the cerebral circulation. The use of brain homogenates in the current study cannot differentiate whether the tau seeds remain in the blood or have disseminated into the brain parenchyma; however, the results indicate that tau in the cerebral circulation may present a risk factor for initiating a brain tauopathy after lung infection.

In summary, our current study resolves (1) the expression of four tau isoforms in lung endothelium; (2) tau expression in lung capillaries *ex vivo*; (3) that virulent strains/mutants of *P. aeruginosa* elicit the release of pathological tau from endothelium that is capable of seeding neural tau aggregates; and (4) that virulent *P. aeruginosa* lung infection promotes a neural tau burden capable of seeding neural tau aggregates. Our results are most consistent with an infectious tauopathy model, wherein lung cells produce a pathological “amyloidogenic” species during infection that is encoded by *MAPT*. In the presence of virulent bacterial pneumonia, these pathological tau species are released and may reach the brain *via* the circulation. Once in the brain, they cause neuronal tau misfolding, aggregation, and propagation, and thereby impair neuronal synaptic transmission and cognitive function. Future studies addressing mechanisms responsible for the dissemination of tau from the lung through the circulation to the brain are needed.

## Experimental procedures

### Cloning lung tau isoforms

Total RNA was isolated from rat lung or rat PMVECs using RNeasy mini kit (Qiagen). After cDNA generation using iScript cDNA synthesis kit (Bio-Rad) with 1 μg RNA in a total volume of 20 μl, rat Tau cDNAs were amplified using Platinum Taq DNA polymerase high fidelity kit (Invitrogen) and rat Tau-specific primers. The primers used for PCR were designed to include restriction enzyme sites for cloning purpose and to remove the stop codon to express a tagged Tau. Forward primer: TAA GCA AGC TT(HindIII)T GAA GCA GCA TGG CTG AAC C and reverse primer: TGC TTA TCT AGA (XbaI) CAA ACC CTG CTT GGC CAA AGA G. The samples were incubated at 95 °C for 3 min, followed by 35 cycles of 95 °C 30 s, 60 °C for 30 s, and 72 °C for 60 s. A final extension step was performed at 72 °C for 5 min. Amplified PCR products were purified, double-digested with HindIII and XbaI, and cloned into the mammalian cell expression vector, pcDNA3.1-V5/His. The cloned rat tau cDNA sequences were verified by bidirectional Sanger sequencing (MCLab). The sequences have been deposited in GenBank (Accession number: 0N4R MZ604975, 1N4R MZ604976, 2N4R MZ604977, and big tau MZ604978).

### Endothelial and bacterial culture and supernatant preparation

Detailed cell/bacteria culture and supernatant preparation procedures have been described previously and is standard for our published studies (16, 17, 18). Briefly, the cell culture core at the University of South Alabama Center for Lung Biology provided rat pulmonary microvascular endothelial cell clones isolated from Sprague–Dawley rats. The cells were cultivated in Dulbecco’s modified Eagle medium (DMEM; 4.5 g/l glucose) with 10% fetal bovine serum (Atlanta Biologicals) and 1% penicillin-streptomycin and incubated at 37 °C with 5% CO_2_.

*P. aeruginosa* isogenic strains PA103 (ExoU/T) has a fully functional type 3 secretion system capable of injecting exoenzymes into the cytoplasmic compartment of the host cell. Conversely, the ΔPcrV (PA103*ΔPCRV*; ExoU and ExoT) mutant lacks the PcrV protein required for a functional type 3 secretion system and is unable to inject exoenzymes into host cells. The ExoY (PA103Δe*xoUexoT*::Tc pUCP*exoY*) mutant is able to both express and secrete the functional ExoY cyclase into target cells. The ExoY^K81M^ (PA103Δe*xoUexoT*::Tc pUCP*exoY*^*K81M*^) mutant expresses a catalytically inactive ExoY that does not generate cyclic nucleotides in host cells. Clinical isolates including PA808 and PA35 were obtained from patients’ bronchoalveolar lavage samples, and *P. aeruginosa* was independently verified by a clinical and genotypes by a research laboratory (32).

For supernatant preparation, PMVECs were first grown to confluence. At 12 to 24 h postconfluence, *P. aeruginosa* from overnight Vogel–Bonner plates were suspended in 1× PBS(Invitrogen) to an A_540_ of 0.25, previously determined to represent 2 × 10^8^ CFU/ml. Endothelial monolayer was infected with bacteria at 5:1 multiplicity of infection in HBSS (Invitrogen) for 3 to 7 h at 37 °C and 5% CO_2_. The supernatants were then collected, centrifuged at 4000*g*, and sterilized *via* passage through a 0.22 μm filter (EMD Millipore). Vehicle control supernatant was generated as described but in the absence of bacteria. The collected supernatants were then precipitated by Trichloroacetic acid including 4 mg/ml of Na-deoxycholate and resuspended in either HBSS (for FRET and BiFC assay) or 1× sample buffer (for SDS-PAGE). One-tenth volume of HBSS or sample buffer was used to resuspend supernatants.

### Cell transfection

HEK293 (human embryonic kidney 293) cells were grown in DMEM supplemented with 10% fetal bovine serum (ATCC) and 1% penicillin-streptomycin and incubated at 37 °C with 5% CO_2_. The transfection of human tau P301L DNA plasmid (plasmid #87634; Addgene) was performed using lipofectamine 3000 (Invitrogen). For transient BiFC studies, the cells were transfected using lipofectamine with pCMV expression plasmid encoding the N-terminus of the Venus fluorescence protein fused to the full-length tau (Vn-tau, plasmid #87368; Addgene) or tau fused to the C-terminus of Venus (tau-Vc, plasmid #87369; Addgene) (47). The transfected cells were used for biomolecular fluorescence complementation studies after 48 h.

For FRET transfection studies, HEK293 cells were plated at a density of ∼50% confluency, and at ∼70% confluence (∼12–18 h), the cells were transfected with endothelium-derived tau seeds prepared from the cell supernatant. Transfection complexes were made by combining [8.75 μl Opti-MEM (Gibco) + 1.25 μl lipofectamine 3000] with [Opti-MEM + (10–20 μl) tau seeds] for a total volume of 20 to 30 μl per well. Liposome preparations were incubated at room temperature for 20 min before adding to the cells. The cells were incubated with transfection complexes for 24 h.

### Generation of *MAPT* knockout cells

*MAPT* knockout (KO) rat PMVECs were generated using CRISPR-Cas9 gene-editing technology, as previously described (48). To successfully knockout the gene that encodes tau in PMVECs, three guide RNA (gRNAs) were used to target Exon 1 of *MAPT* gene: (1) 5′-ATTGTGTCAAACTCCTGGCG; (2) 5′-GTTTGACACAATGGAAGACC; and (3) 5′-GCATAGTGTAATCTCCGGCC. A day before transfection, PMVECs were seeded on 24-well plate with 7.5 × 10^4^ per well. The gRNA, CRISPR RNA, and transactivating crRNA and Cas9 enzymes (Invitrogen) were incubated to form a Ribonucleoprotein complex. Transfection of ribonucleoprotein complex into PMVECs was performed using Lipofectamine RNAiMAX (Invitrogen). Two days after transfection, genomic DNAs were isolated and used to do cleavage assay using GeneArt Genomic Cleavage detection kit (Life Technologies) and two primers: forward ATGTCACCTGCTTTAGTGGG, and reverse AATCTAGGATTTGGGGCTGG. Cleavage assay results showed that the gRNA2 most efficiently modified genomic DNA; thus, gRNA2-transfected cells were single-cell sorted using fluorescence-activated cell sorting (BD Bioscience). The targeted genomic loci in isolated genomic DNA from these clones were amplified by PCR and inserted into the pGEM-T vector (Promega). Ten to 15 plasmids from each KO candidate were subjected to DNA sequencing and verification. The clones showing frameshift mutations in all sequences were selected as a *MAPT* KO cells and used for experiments.

### Generation of stable BiFC cells

HEK293 cells were transfected using lipofectamine with plasmids encoding Vn-tau, tau-Vc, or with both plasmids. The cells were grown for 48 h, and the medium was replaced with the selecting media containing 500 μg/ml of Neomycin (Gibco). Resistant cells were further expanded, harvested, resuspended in flow cytometry buffer (HBSS plus 1% fetal bovine serum and 1 mM EDTA), and subjected to fluorescence-activated cell sorting. Nontransfected, Vn, or Vc positive cells were used to set the gating parameters. Populations of Vn and Vc dual positive cells were selected into 96-well plates for single-cell clones. The Vn-tau and tau-Vc expression levels in the single cell clones were determined using Western blotting, and clones with a ∼1:1 ratio were selected and used for the BiFC studies.

### FRET measurement

Transfected HEK293 cells (CRL 3275; ATCC) were imaged using laser confocal microscopy. FRET images were acquired using a Nikon A1R confocal microscope equipped with 20× water immersion objective (Plan Apo VC 20× DIC WI WD-1.00 NA-0.75; Nikon Instruments). The images were acquired using excitation wavelength at 405 nm to excite the donor CFP and emission wavelength at 535 nm from the acceptor YFP. Quantification of the images was performed using FIJI ImageJ and pixFRET, as detailed in the previous studies (49). Briefly, unless otherwise stated, the three different settings used for the analysis of FRET with the CFP/YFP pair were as follows: (1) FRET: Ex 405 nm/Em 535 nm; (2) CFP: Ex 405 nm/Em 475 nm; and (3) YFP: Ex 514 nm/Em 535 nm. Laser power and detector gain were initially optimized and kept unchanged for all samples. Linear and exponential fits were performed in pixFRET, and FRET measured was corrected for spectral bleed-throughs, normalized for expression levels, after the pixFRET manual and as previously described (49). FRET signals from each set of the study were normalized to that of the vehicle controls performed in parallel.

### BiFC measurement

HEK293 cells stably expressing BiFC probes were used. Cells grown at ∼70% confluence on 12-well plate containing coverslips were either incubated with 8-pCPT-cAMP (15 μM), 8-pCPT-cGMP (15 μM), okadaic acid (4 nM), or vehicle (DMSO), or were transfected with endothelium-derived tau seeds prepared from the control or bacteria-infected cell supernatant using the Xfect protein transfection reagent (Clontech Lab) according to manufacturer’s instruction. Briefly, 6 μl of Xfect reagent diluted in 34 μl of water was mixed with 10 to 20 μl of supernatant diluted in 20 to 30 μl of Xfect protein buffer and incubated for 30 min at room temperature (for a final volume of 80 μl per sample). During the mixture incubation, the cells were washed with warm PBS, and 220 μl of opti-MEM was added to each well containing cells. The 80 μl transfection reagent/supernatant mixture was added to the cells in 220 μl opti-MEM and incubated at 37 °C for 60 min. One milliliter of complete growth medium (*i.e.*, containing 10% fetal bovine serum) was then added to continue incubation overnight for BiFC assay. Coverslips were mounted after cell fixation in 4% paraformaldehyde solution for 15 min at room temperature. Acquisition of the fluorescence signal was performed using a Nikon A1R confocal microscopy and the 20× water immersion objective, as described above. The fluorescence of Venus protein was acquired using excitation wavelength at 514 nm and emission wavelength at 535 nm. The cellular intensities of tau-BiFC fluorescence were analyzed using FIJI-ImageJ. From each fluorescence micrographs, the fraction of cell area with high fluorescence intensity was calculated from the following: baseline fluorescence signal was determined using a low thresholding parameter (to get the total cell area) and higher intensity fluorescence signal was determined using vehicle control’s thresholding parameters (to get the cell area with high fluorescence signal). For each experimentation, tau-BiFC signal was expressed as a percentage by normalizing the fraction of cell area with high fluorescence intensity to the fraction of cell area from vehicle control performed in parallel.

### Western blot

Tissue and Cell lysates were prepared in RIPA buffer. The harvested lung tissue was cleaned in cold PBS, snap freeze in liquid nitrogen, and stored at −80 °C until use. Frozen tissues were pulverized in the chilled pulverizer in liquid nitrogen (Cole-Parmer). 30∼50 mg of pulverized tissue powder then was homogenized in 400∼500 μl of RIPA buffer containing 2 mM EDTA and protease inhibitor cocktail using an ultra-turax homogenizer (Tekmar) on ice. After centrifugation at 14,500 rpm for 15 min at 4 °C, the supernatant was transferred to new tubes, followed by BCA assay (Thermo Scientific) to determine protein concentration. The proteins were transferred onto nitrocellulose membrane (Bio-Rad) after SDS-PAGE using Bolt 4∼12% Bis-Tris Plus gel (Invitrogen). After blocking in 5% fat-free milk in TBS/T for an hour at room temperature, primary antibodies were incubated at 4 °C overnight. The used primary antibodies were D1M9X (Cell Signaling Technology, 1:2000), Tau-5 (MBL, 1:2000), TNT-1 (EMD, 1:2000), and Actin (BD Biosciences, 1:5000). Appropriate HRP-conjugated secondary antibodies were incubated, and the supersignal chemiluminescent substrate (Thermo Scientific) was used to detect proteins.

### Animals and infection

Experimentation with animals was approved by the Institutional Animal Care and Use Committee of the University of South Alabama and conducted according to the “Guide to the Care and Use of Laboratory Animals.” Adult WT (12 weeks-old; C57BL/6J) and tau knockout (B6.129S4(Cg)-*Mapt*^*tm1(EGFP)/Klt*^/J, Stock No: 029219; The Jackson Laboratory) mice were used. For primary infection studies, the mice were anesthetized by intraperitoneal injection of a ketamine/xylazine mixture (80/5 mg/kg of body weight) and inoculated intratracheally with ExoY (10^5^ CFU in 40 μl) or saline vehicle (40 μl). At 48 h after the infection, the brains were collected immediately after euthanasia, snap-frozen, and pulverized (Cole-Parmer) in liquid nitrogen to tissue powder. Subsequently, 30 to 50 mg of pulverized tissues were homogenized in 300 μl of HBSS containing 2 mM EDTA and protease inhibitor cocktail using an ultra-turax homogenizer (Tekmar) at 4 °C. After centrifugation at 14,500 rpm for 15 min at 4 °C, the supernatant was transferred to new tubes, followed by protein concentration determination and preparation as tau seeds for transfection and BiFC studies. The transfection of brain homogenate was performed with Xfect protein transfection reagent, and 5 μg of the brain homogenate were used, as described above.

### Lung preparation and fluorescence imaging

We used the agarose/gelatin-infused lung slice technique to expand air sacs and vessels to evaluate eGFP expression, tomato lectin Dylight594 (Vector Laboratories) vascular staining, and nuclear staining (DAPI for live cell, Invitrogen), as we have previously described (30). The mice were deeply sedated with an injection of ketamine/xylazine mixture. Heparin was also injected together with the anesthetics at a dose of 5000 IU/kg body weight, followed by a retro-orbital injection of 70 μl (70 μg) tomato lectin and tracheal cannulation using a 22G tubing. After thoracotomy, another dose of 3 IU heparin was injected directly into the right ventricle. A plastic cannula connected to a reservoir was advanced into the pulmonary artery *via* an incision in the right ventricular free wall, and the cannula was secured with a suture. Next, a second plastic cannula of the same size was advanced into the left atrium *via* an incision in the apex of the left ventricle. Both cannulas were secured by a cotton thread tied around the ventricles. HBSS (15 ml) was slowly perfused through the pulmonary circulation with a differential gravity of 40 cmH_2_O, followed by a 6% warm gelatin/HBSS (Thermo Fisher Scientific) solution (∼10 ml). On completion of lung perfusion, both the cannulas were clamped with hemostats. Next, 0.6 ml of 2% warm agarose/HBSS (low melting point; Thermo Fisher Scientific) solution was infused through the trachea to inflate the lung. Ice was used *in situ* to solidify the gel *en bloc* for 30 min before the lung was harvested. The left lung was sliced into 800 μm transverse slices on a vibratome (Leica Biosystems), incubated in DAPI for 20 min at room temperature, washed, and imaged using a Nikon A1R multiphoton microscope. The integrated densities of eGFP signal of at least five random representative images from the lung were quantified using ImageJ.

### Data and analysis

Offline data analysis and statistical comparison were performed using Excel (Microsoft), Igor Pro (WaveMatrics), and Prism (GraphPad), in addition to the already specified quantification software and packages. For FRET and BiFC studies, triplicates were performed, and each experiment (n) represents averaged signal from the triplicate measurements. For animal studies, each experimentation (n) indicates the number of mice; BiFC signal was quantified from triplicate measurements. The data are expressed as mean ± SD, or as specified. Statistical comparisons were performed using ANOVA followed by Dunnett’s *post hoc* test, where *p* < 0.05 was denoted as statistically significant.

## Data availability

All data are contained within the article.

## Conflict of interest

The authors declare that they have no conflicts of interest with the contents of this article.

## References

[1] Ballatore C., Lee V.M., Trojanowski J.Q. (2007). Tau-mediated neurodegeneration in Alzheimer's disease and related disorders. Nat. Rev. Neurosci..

[2] Braak H., Braak E. (1991). Neuropathological stageing of Alzheimer-related changes. Acta Neuropathol..

[3] Ittner L.M., Gotz J. (2011). Amyloid-beta and tau--a toxic pas de deux in Alzheimer's disease. Nat. Rev. Neurosci..

[4] Mandelkow E.M., Mandelkow E. (2012). Biochemistry and cell biology of tau protein in neurofibrillary degeneration. Cold Spring Harb. Perspect. Med..

[5] Jucker M., Walker L.C. (2013). Self-propagation of pathogenic protein aggregates in neurodegenerative diseases. Nature.

[6] Lace G., Savva G.M., Forster G., de Silva R., Brayne C., Matthews F.E., Barclay J.J., Dakin L., Ince P.G., Wharton S.B., Mrc C. (2009). Hippocampal tau pathology is related to neuroanatomical connections: An ageing population-based study. Brain.

[7] Frost B., Jacks R.L., Diamond M.I. (2009). Propagation of tau misfolding from the outside to the inside of a cell. J. Biol. Chem..

[8] Frost B., Ollesch J., Wille H., Diamond M.I. (2009). Conformational diversity of wild-type tau fibrils specified by templated conformation change. J. Biol. Chem..

[9] Wang Y., Balaji V., Kaniyappan S., Kruger L., Irsen S., Tepper K., Chandupatla R., Maetzler W., Schneider A., Mandelkow E., Mandelkow E.M. (2017). The release and trans-synaptic transmission of tau via exosomes. Mol. Neurodegener..

[10] Laurent C., Buee L., Blum D. (2018). Tau and neuroinflammation: What impact for Alzheimer's disease and tauopathies?. Biomed. J..

[11] Liu L., Drouet V., Wu J.W., Witter M.P., Small S.A., Clelland C., Duff K. (2012). Trans-synaptic spread of tau pathology *in vivo*. PLoS One.

[12] Sagare A.P., Bell R.D., Zhao Z., Ma Q., Winkler E.A., Ramanathan A., Zlokovic B.V. (2013). Pericyte loss influences Alzheimer-like neurodegeneration in mice. Nat. Commun..

[13] Rauch J.N., Luna G., Guzman E., Audouard M., Challis C., Sibih Y.E., Leshuk C., Hernandez I., Wegmann S., Hyman B.T., Gradinaru V., Kampmann M., Kosik K.S. (2020). LRP1 is a master regulator of tau uptake and spread. Nature.

[14] He Z., McBride J.D., Xu H., Changolkar L., Kim S.J., Zhang B., Narasimhan S., Gibbons G.S., Guo J.L., Kozak M., Schellenberg G.D., Trojanowski J.Q., Lee V.M. (2020). Transmission of tauopathy strains is independent of their isoform composition. Nat. Commun..

[15] Holmes B.B., Furman J.L., Mahan T.E., Yamasaki T.R., Mirbaha H., Eades W.C., Belaygorod L., Cairns N.J., Holtzman D.M., Diamond M.I. (2014). Proteopathic tau seeding predicts tauopathy *in vivo*. Proc. Natl. Acad. Sci. U. S. A..

[16] Ochoa C.D., Alexeyev M., Pastukh V., Balczon R., Stevens T. (2012). *Pseudomonas aeruginosa* exotoxin Y is a promiscuous cyclase that increases endothelial tau phosphorylation and permeability. J. Biol. Chem..

[17] Morrow K.A., Ochoa C.D., Balczon R., Zhou C., Cauthen L., Alexeyev M., Schmalzer K.M., Frank D.W., Stevens T. (2016). *Pseudomonas aeruginosa* exoenzymes U and Y induce a transmissible endothelial proteinopathy. Am. J. Physiol. Lung Cell. Mol. Physiol..

[18] Balczon R., Morrow K.A., Zhou C., Edmonds B., Alexeyev M., Pittet J.F., Wagener B.M., Moser S.A., Leavesley S., Zha X., Frank D.W., Stevens T. (2017). *Pseudomonas aeruginosa* infection liberates transmissible, cytotoxic prion amyloids. FASEB J..

[19] Pandharipande P.P., Girard T.D., Jackson J.C., Morandi A., Thompson J.L., Pun B.T., Brummel N.E., Hughes C.G., Vasilevskis E.E., Shintani A.K., Moons K.G., Geevarghese S.K., Canonico A., Hopkins R.O., Bernard G.R. (2013). Long-term cognitive impairment after critical illness. N. Engl. J. Med..

[20] Iwashyna T.J., Ely E.W., Smith D.M., Langa K.M. (2010). Long-term cognitive impairment and functional disability among survivors of severe sepsis. JAMA.

[21] Shah F.A., Pike F., Alvarez K., Angus D., Newman A.B., Lopez O., Tate J., Kapur V., Wilsdon A., Krishnan J.A., Hansel N., Au D., Avdalovic M., Fan V.S., Barr R.G. (2013). Bidirectional relationship between cognitive function and pneumonia. Am. J. Respir. Crit. Care Med..

[22] Balczon R., Lin M.T., Lee J.Y., Abbasi A., Renema P., Voth S.B., Zhou C., Koloteva A., Michael Francis C., Sodha N.R., Pittet J.F., Wagener B.M., Bell J., Choi C.S., Ventetuolo C.E. (2021). Pneumonia initiates a tauopathy. FASEB J..

[23] Goedert M., Spillantini M.G., Cairns N.J., Crowther R.A. (1992). Tau proteins of Alzheimer paired helical filaments: Abnormal phosphorylation of all six brain isoforms. Neuron.

[24] Lee V.M., Goedert M., Trojanowski J.Q. (2001). Neurodegenerative tauopathies. Annu. Rev. Neurosci..

[25] Goedert M., Spillantini M.G., Crowther R.A. (1992). Cloning of a big tau microtubule-associated protein characteristic of the peripheral nervous system. Proc. Natl. Acad. Sci. U. S. A..

[26] Gu Y., Oyama F., Ihara Y. (1996). Tau is widely expressed in rat tissues. J. Neurochem..

[27] Mandelkow E., von Bergen M., Biernat J., Mandelkow E.M. (2007). Structural principles of tau and the paired helical filaments of Alzheimer's disease. Brain Pathol..

[28] Vanlandewijck M., He L., Mae M.A., Andrae J., Ando K., Del Gaudio F., Nahar K., Lebouvier T., Lavina B., Gouveia L., Sun Y., Raschperger E., Rasanen M., Zarb Y., Mochizuki N. (2018). A molecular atlas of cell types and zonation in the brain vasculature. Nature.

[29] He L., Vanlandewijck M., Mae M.A., Andrae J., Ando K., Del Gaudio F., Nahar K., Lebouvier T., Lavina B., Gouveia L., Sun Y., Raschperger E., Segerstolpe A., Liu J., Gustafsson S. (2018). Single-cell RNA sequencing of mouse brain and lung vascular and vessel-associated cell types. Sci. Data.

[30] Alexeyev M., Geurts A.M., Annamdevula N.S., Francis C.M., Leavesley S.J., Rich T.C., Taylor M.S., Lin M.T., Balczon R., Knighten J.M., Alvarez D.F., Stevens T. (2020). Development of an endothelial cell-restricted transgenic reporter rat: A resource for physiological studies of vascular biology. Am. J. Physiol. Heart Circ. Physiol..

[31] Balczon R., Morrow K.A., Leavesley S., Francis C.M., Stevens T.C., Agwaramgbo E., Williams C., Stevens R.P., Langham G., Voth S., Cioffi E.A., Weintraub S.E., Stevens T. (2020). Cystatin C regulates the cytotoxicity of infection-induced endothelial-derived beta-amyloid. FEBS Open Bio.

[32] Voth S., Gwin M., Francis C.M., Balczon R., Frank D.W., Pittet J.F., Wagener B.M., Moser S.A., Alexeyev M., Housley N., Audia J.P., Piechocki S., Madera K., Simmons A., Crawford M. (2020). Virulent Pseudomonas aeruginosa infection converts antimicrobial amyloids into cytotoxic prions. FASEB J..

[33] Lin M.T., Balczon R., Pittet J.F., Wagener B.M., Moser S.A., Morrow K.A., Voth S., Francis C.M., Leavesley S., Bell J., Alvarez D.F., Stevens T. (2018). Nosocomial pneumonia elicits an endothelial proteinopathy: Evidence for a source of neurotoxic amyloids in critically ill patients. Am. J. Respir. Crit. Care Med..

[34] Balczon R., Pittet J.F., Wagener B.M., Moser S.A., Voth S., Vorhees C.V., Williams M.T., Bridges J.P., Alvarez D.F., Koloteva A., Xu Y., Zha X.M., Audia J.P., Stevens T., Lin M.T. (2019). Infection-induced endothelial amyloids impair memory. FASEB J..

[35] Scott A.M., Jager A.C., Gwin M., Voth S., Balczon R., Stevens T., Lin M.T. (2020). Pneumonia-induced endothelial amyloids reduce dendritic spine density in brain neurons. Sci. Rep..

[36] Kfoury N., Holmes B.B., Jiang H., Holtzman D.M., Diamond M.I. (2012). Trans-cellular propagation of tau aggregation by fibrillar species. J. Biol. Chem..

[37] Lewis J., McGowan E., Rockwood J., Melrose H., Nacharaju P., Van Slegtenhorst M., Gwinn-Hardy K., Paul Murphy M., Baker M., Yu X., Duff K., Hardy J., Corral A., Lin W.L., Yen S.H. (2000). Neurofibrillary tangles, amyotrophy and progressive motor disturbance in mice expressing mutant (P301L) tau protein. Nat. Genet..

[38] Kerppola T.K. (2006). Design and implementation of bimolecular fluorescence complementation (BiFC) assays for the visualization of protein interactions in living cells. Nat. Protoc..

[39] Tak H., Haque M.M., Kim M.J., Lee J.H., Baik J.H., Kim Y., Kim D.J., Grailhe R., Kim Y.K. (2013). Bimolecular fluorescence complementation; lighting-up tau-tau interaction in living cells. PLoS One.

[40] Wagener B.M., Anjum N., Christiaans S.C., Banks M.E., Parker J.C., Threet A.T., Walker R.R., Isbell K.D., Moser S.A., Stevens T., Alexeyev M.F., Audia J.P., Richter W., Hardy K.S., Saleh L.A. (2020). Exoenzyme Y contributes to end-organ dysfunction caused by Pseudomonas aeruginosa pneumonia in critically ill patients: An exploratory study. Toxins (Basel).

[41] Trojanowski J.Q., Schuck T., Schmidt M.L., Lee V.M. (1989). Distribution of tau proteins in the normal human central and peripheral nervous system. J. Histochem. Cytochem..

[42] Boyne L.J., Tessler A., Murray M., Fischer I. (1995). Distribution of big tau in the central nervous system of the adult and developing rat. J. Comp. Neurol..

[43] Fischer I., Baas P.W. (2020). Resurrecting the mysteries of big tau. Trends Neurosci..

[44] Stevens T.C., Ochoa C.D., Morrow K.A., Robson M.J., Prasain N., Zhou C., Alvarez D.F., Frank D.W., Balczon R., Stevens T. (2014). The *Pseudomonas aeruginosa* exoenzyme Y impairs endothelial cell proliferation and vascular repair following lung injury. Am. J. Physiol. Lung Cell. Mol. Physiol..

[45] Mukrasch M.D., Biernat J., von Bergen M., Griesinger C., Mandelkow E., Zweckstetter M. (2005). Sites of tau important for aggregation populate {beta}-structure and bind to microtubules and polyanions. J. Biol. Chem..

[46] Li W., Lee V.M. (2006). Characterization of two VQIXXK motifs for tau fibrillization *in vitro*. Biochemistry.

[47] Blum D., Herrera F., Francelle L., Mendes T., Basquin M., Obriot H., Demeyer D., Sergeant N., Gerhardt E., Brouillet E., Buee L., Outeiro T.F. (2015). Mutant huntingtin alters tau phosphorylation and subcellular distribution. Hum. Mol. Genet..

[48] Lee J.Y., Alexeyev M., Kozhukhar N., Pastukh V., White R., Stevens T. (2018). Carbonic anhydrase IX is a critical determinant of pulmonary microvascular endothelial cell pH regulation and angiogenesis during acidosis. Am. J. Physiol. Lung Cell. Mol. Physiol..

[49] Feige J.N., Sage D., Wahli W., Desvergne B., Gelman L. (2005). PixFRET, an ImageJ plug-in for FRET calculation that can accommodate variations in spectral bleed-throughs. Microsc. Res. Tech..

